# Automatic Identification of Hate Speech – A Case-Study of alt-Right YouTube Videos

**DOI:** 10.12688/f1000research.147107.1

**Published:** 2024-04-23

**Authors:** Johan Eddebo, Mika Hietanen, Mathias Johansson

**Affiliations:** 1 Centre for Multidisciplinary Research on Religion and Society (CRS), Uppsala University, Uppsala, Sweden; 2 Department of Communication and Media, Lund University, Lund, 221 00, Sweden; 3 Department of Arts and Cultural Sciences, Lund University, Lund, Sweden

**Keywords:** Automatic hate speech identification; Hate speech; alt-right; YouTube; interdisciplinary research

## Abstract

**Background:**

Identifying hate speech (HS) is a central concern within online contexts. Current methods are insufficient for efficient preemptive HS identification. In this study, we present the results of an analysis of automatic HS identification applied to popular alt-right YouTube videos.

**Methods:**

This essay describes methodological challenges of automatic HS detection. The case study concerns data on a formative segment of contemporary radical right discourse. Our purpose is twofold. (1) To outline an interdisciplinary mixed-methods approach for using automated identification of HS. This bridges the gap between technical research on the one hand (such as machine learning, deep learning, and natural language processing, NLP) and traditional empirical research on the other. Regarding alt-right discourse and HS, we ask: (2) What are the challenges in identifying HS in popular alt-right YouTube videos?

**Results:**

The results indicate that effective and consistent identification of HS communication necessitates qualitative interventions to avoid arbitrary or misleading applications. Binary approaches of hate/non-hate speech tend to force the rationale for designating content as HS. A context-sensitive qualitative approach can remedy this by bringing into focus the indirect character of these communications. The results should interest researchers within social sciences and the humanities adopting automatic sentiment analysis and for those analysing HS and radical right discourse.

**Conclusions:**

Automatic identification or moderation of HS cannot account for an evolving context of indirect signification. This study exemplifies a process whereby automatic hate speech identification could be utilised effectively. Several methodological steps are needed for a useful outcome, with both technical quantitative processing and qualitative analysis being vital to achieve meaningful results. With regard to the alt-right YouTube material, the main challenge is indirect framing. Identification demands orientation in the broader discursive context and the adaptation towards indirect expressions renders moderation and suppression ethically and legally precarious.

The identification of hate speech is a central concern within online contexts. Current regulation guidelines require that platforms address material flagged as hate speech within 24 hours ( Hern, 2016; European Commission, 2019). Automatic identification has the potential to flag content before users are exposed to it, thereby lessening its harmfulness. As the amount of user generated content grows, the amount of hate speech grows with it as well as the needs for automatic identification ( Basile et al., 2019, p. 54).

Current methods are insufficient to enable efficient pre-emptive hate speech identification. Although central within current major challenges for online platforms, the concept of hate speech has not even been clearly defined. Definitions commonly mention such communications that disparage a person or a group based on such general protected characteristics as ‘race, color, national origin, sex, disability, religion or sexual orientation’ ( Nockleby, 2000, pp. 1277–1279) and the big tech platforms have presented more specific lists of forbidden types of communication, but they are not uniform (for a discussion, see Hietanen & Eddebo, 2023). Beyond the issue of how to define hate speech, lie moral and ethical considerations of *algorithmic censorship* ( Cobbe, 2021). Regardless, there are numerous studies on the identification of hate speech and hate speech-adjacent communication in online environments, such as toxicity ( d’Sa et al., 2020; Maslej-Krešňáková et al., 2020), misogyny ( Farrell et al., 2019), offensive language ( Zampieri et al., 2019), personal attacks ( Wulczyn et al., 2017). These types of studies are typically focused on the tools and performances over the phenomenon and its characteristics ( Dowlagar & Mamidi, 2021; Badjatiya et al., 2017; Alonso et al., 2020). On the other hand, empirically oriented studies of hate speech (and similar) typically employ a *distant reading* approach, providing a scant overview of the phenomenon rather than studying the material itself ( Farrell et al., 2019; Paasch-Colberg et al., 2021). As an example, Ottoni et al. (2018) have studied vocabulary and biases of right-wing YouTube content creators in conjunction with the comment sections. Such a study can illuminate broader tendencies, such as that right wing channels are more likely to discuss war and terrorism, topics with strong negative associations. However, little attention is paid to the narratives or specific uses of language beyond the lexical.

In this study, we present the results of an analysis of automatic hate speech identification in popular alt-right YouTube videos. Our purpose is twofold, and regards methodology as well as the analysis of the character of contemporary hate speech discourse, especially in terms of detection-evasive strategies as exemplified by the alt-right. From the point of view of method, we wish, (1) for social science and humanities researchers, to outline a mixed-methods approach for using automated identification of hate speech in the digital sphere by transparently accounting for the steps needed when utilising automatic hate speech identification, including key challenges in the process. This is intended to bridge the gap between an, often, technical type of research on the one hand (such as machine learning, ML, deep learning, DL, and natural language processing, NLP) and traditional empirical research on the other. Regarding the character of alt-right discourse and hate speech in particular, we ask: (2) What are the challenges in identifying hate speech in popular alt-right YouTube videos that are intentionally designed to circumvent filters and regulations? The results should be of interest for researchers within social sciences and the humanities adopting automatic sentiment analysis as part of their methods of analysis in general, and for those analysing hate speech and radical right discourse specifically.

## Methods

### Overview

There is no cut-and-ready method for our purposes. By necessity our approach is experimental in design and should be seen as exploratory when it comes to how a verbal and contextual phenomenon such as hate speech can be identified automatically. Our twofold purpose has led us to implement two complementary methodological approaches. In the following, we first describe an approach to automatic identification in four steps: gathering an annotated corpus (for this we have used three corpora); text pre-processing; training of the classifier and applying it to the YouTube transcripts. Finally, we engage with the material through a qualitative close reading of select parts to better understand the previous step, and the character of the material in general.

### Automatic identification of hate speech

The challenge of automatically classifying the sentiment of a text has a long history ( Biagioni, 2016), and the sub-problem of identifying what is offensive in texts has also been given attention ( Schmidt & Wiegand, 2019). A methodologically simple method is the lexical approach: to rely on an annotated lexicon of terms related to the sentiment (positively and negatively) and to count the frequencies of terms in a text. If there are more positive (or negative) terms or phrases in the text it can be classified as positive (or negative). This has some obvious drawbacks: terms are used variously and can relate to a context in contradictory ways. An example of this is *sarcasm.* A statement or phrasing that uses a term in its non-literal sense is a challenge for language-modelling. This is especially true when such statements are also used in their literal meaning in the same corpus. Hate speech is a specific variation of sentiment analysis; instead of classifying statements on a scale between two opposites (positive and negative) it classifies statements as hate speech or not hate speech. Gitari et al. (2015) perform a series of tests using this lexical approach to the issue of hate speech, with limited success.

With the advancement of machine learning (ML) and deep learning (DL) methods, the sophistication and range of available methods have increased. These methods tend to share one typical drawback: they are data hungry, that is, they need a lot of data to be trained to perform their task ( Ploeg et al., 2014; Adadi, 2021). Typically, the more data and varied data a model is trained on, the better it will generalise what it learns to new data. When data is not readily available, collecting this type of large-scale data is time-consuming, especially if it needs to be annotated. When just having a lot of data is not enough or the required classification is not available it is standard practice to rely on manual annotation. It has become common to crowdsource this task, using volunteers or commissioned individuals using platforms such as *Zooniverse* and *Figure 8* ( Wang *et al*., 2013).

### Data for training the model

For this study we combined three openly available hate speech annotated corpora of tweets written in English: Hate-speech-and-offensive-language, HSOL ( Davidson et al., 2017), *HatEval* – as subset of the HatEval corpus ( Basile et al., 2019), which in turn is based on a corpus for misogyny identification by Fersini et al., (2019), and Offensive Language Identification Dataset, OLID ( Zampieri et al., 2019). These corpora were created with different purposes in mind and their definitions of hate speech are similar but not identical. This is not a problem because together they provide a holistic view of what people consider hate speech.

The annotation of each corpus was achieved through crowdsourcing – enlisting crowds of people to manually annotate tweets – using a rule of majority to determine each tweet’s de facto category. While this is an effective way to get each tweet annotated by multiple people for large corpora and reducing the bias of the individual annotator, it also adds some opacity to the process. The annotators are anonymous and only one of the three corpora has published the instructions provided to the annotators ( Basile, n.d.). While human annotation is considered the golden standard, it is not perfect. Davidson et al. (2017) noted that annotators had more disagreement of what should be regarded as hate speech and what is merely offensive than what is *neutral*; an observation that reflected in the performance of their model. As our purpose is to make a binary classification between hate speech and not, we discarded tweets belonging to the other categories from each corpus and kept a total of 42,430 tweets from the three corpora that were annotated as hate speech and neutral.

### Towards a classifying model

Using this corpus of 42,430 tweets we proceeded to train a classifier, the first part of which is a simple preprocessing pipeline that transforms the raw text into numeric input for the actual model as follows: (1) cleaning, (2) tokenizing, (3) stemming, (4) removing stop words, and (5) vectorizing. Out of these five steps only the cleaning needs to be customised, which is why it will be explained in more detail in the next subsection.

The first step, explained in more detail below, homogenises from the raw text format to a lowercase string of characters. These strings are then split into their constituent terms, *tokens.* These tokens are then stemmed, i.e., reduced to their core meaning or word by removing endings. This step may reduce different meaning tokens to a similar token, such as the verb ‘booking’ and the noun ‘books,’ which both become ‘book.’ This step reduces the number of tokens dramatically and reduces the chance that the model learns too much from a rarely used variant of a word.

From these tokens we remove words that are too common to add specific meanings to the texts, words like ‘a,’ ‘of,’ ‘and.’ The tokens that remain after this step make the basis for the model’s vocabulary, the ‘words’ or tokens the model will recognize.

### Data cleaning and preparation

Special attention was paid to cleaning the tweets, since their format is different compared to our empirical material of transcriptions from YouTube. There are some conventions of Twitter and other social media that do not translate well to other written media and not at all to spoken work. For example, the use of @USER to direct one’s statement to a particular user, the inclusion of URLs, the use of emojis, and of hashtags. The latter sometimes consist of single words, a phrase, or an acronym, which sometimes can easily be separated automatically into its parts. None of these conventions exist in transcriptions and were therefore removed. The output of this step is a lowercase string of characters without @ or # tags, and without URLs.

### Training the hate speech classifier

For the training and selection of models we used the *SciKitLearn* and *Tensorflow* (Keras) libraries, as these provide a lot of stable and efficient algorithms for ML models and selection. Before vectorizing and standardising the corpus, we split the data 80:20 randomly stratified by the outcome variable, depending on if the tweet was annotated as hate speech or not, to ensure that we have a similar distribution of hate speech and non-hate speech in both segments. The 20% was withheld from the model during training and was at the end used to evaluate the model. This reduces overfitting, that the model learns too much from the specific dataset it was trained on and therefore cannot generalise to new data. Similarly, some ML and DL algorithms withhold parts of the training data during the training for similar effect.

After experimenting with simpler models in *SciKitLearn*, random forests ( Breiman, 2001), logistic regression ( Menard, 2010), and Naïve Bayes, ( Manning et al., 2008), which did not yield satisfactory results, we moved on to working with *TensorFlow* to train Artificial Neural Network (ANN) models. Specifically, we used Convolutional Neural Networks, CNN ( LeCun et al., 1990) and Recurrent Neural Networks, RNN ( Hochreiter & Schmidhuber, 1997; both explained in Kotu & Deshpande, 2018, ch. 10) which are more complex in nature and have the potential to solve more complex problems. We used *TensorFlow’s* ‘hyperband tuning’ feature to select the final RNN architecture with a testing performance of f-1 score of .854.

### Output

The end-product of this process is a trained classifier that takes texts as an input and returns a value within the range of [0,1] which can be interpreted as an intensity or probability of hate speech within that text. Table 1 contains the confusion matrix of the model’s predictions based on the testing data, the 20% of the corpus withheld during training. We can see that the model manages to correctly classify 89% of the hate speech tweets as hate speech but only identifies 74% of the non-hate speech tweets as non-hate speech. We can therefore expect it to reliably predict when a text does not contain hate speech, but we should be more sceptical when a text is predicted as hate speech.

**Table 1.** Confusion matrix of the trained model’s performance on the testing set.

Annotation	Predicted	
	Hate	No hate
Hate	89	11
No hate	26	74

Note. Percentages are calculated by annotated categories.

### Structuring transcriptions for classification

The limit imposed on tweets is on character level, namely 280 characters (since 2017; earlier 140 characters; Rosen & Ihara, 2017). Roughly speaking, this restricts tweets to at most three sentences. Simultaneously, there is no technical restriction for how long a sentence can be and our transcripts lack punctuation.

Working from the concept of sentences we segmented the transcripts into synthetic sentences by dividing the texts into atoms of seven words and joining every three consecutive atoms into sentences. In this way we have 21-word sentences and atoms are included in three sentences, see Table 2.

**Table 2.** Structure of synthetic sentences.

Sentence _0_: [	Atom _0_	Atom _2_	Atom _3_	]		
	Sentence _1_: [	Atom _2_	Atom _3_	Atom _4_	]	
		Sentence _2_: [	Atom _3_	Atom _4_	Atom _5_	]

We overlapped the sentences to reduce the chances that we split a spoken sentence in ways that remove words from their context. The three-atom schema is the smallest combination that ensures that each atom is at the centre of the selected context. We experimented with different atom-lengths on a few different pieces of the material and inspected the results manually before deciding on the 7-word atom. With shorter atoms, many phrases that manually were marked as hate speech, were not identified as such by the algorithm and longer atoms did not improve results.

When looking at the transcripts on paragraph level we used a similar approach to aggregate the synthetic sentences into synthetic paragraphs using seven atoms around a paragraph’s central atom using the earlier output from the classifier for full synthetic sentences. As illustrated by Table 3, the first and last atom from each video transcript is only used in one synthetic paragraph. Even so, the central atoms of each paragraph are used up to three times due to how we overlapped the paragraphs.

**Table 3.** Structure of synthetic paragraphs.

Par _0_: [	Sen _0_	…	Sen _7_	…	Sen _14_	]				
		Par _1_: [	Sen _7_	…	Sen _14_	…	Sen _21_	]		
			Par _2_: [	…	Sen _14_	…	Sen _21_	…	Sen _28_	]

For the overall score of each paragraph, we used the mean, median and maximum scores of all full synthetic sentences of the paragraph as independent probabilities of the paragraphs containing hate speech and calculated their complement as a measurement for the likelihood that the paragraph contains hate speech.

### Close reading

The outcome of the automated identification is evaluated (below) through a close reading approach, discerning the explicit and implicit argumentative structure and significations ( Brummett, 2010). The material flagged as likely to be hate speech, as well as the low-probability material, are furthermore scrutinised in relation to a holistic definition of hate speech as well as the textual context as such.

We base our understanding of hate speech on the discussion in Hietanen & Eddebo (2023) which refers to speech acts or acts of communication which express intended harm, disparagement, or vilification, or inherently imply the same, and target a group or set of groups defined in relation to protected characteristics, such as race, gender, or religion (for lists of protected characteristics, see Table 2 in Hietanen & Eddebo, 2023).

## Material: Alt-Right on YouTube

### YouTube Alt-Right Corpus

Our selection of channels and videos for analysis is based on Data & Society’s report *Alternative Influence* ( Lewis, 2018) which maps networks of influencers on YouTube characterised by ‘reactionary’ positions, ‘a general opposition to feminism, social justice, or left-wing politics’ (p. 8). The report presents an overview of channels with right-wing politics as well as a positive approach in relation to the radical right or alt-right movement. The YouTube transcripts, automated or uploaded by the channel, were downloaded as text-files. A manual check indicated that the quality of the transcripts was high and reliably conveyed the oral narrative.

We further selected a number of channels formally connected to the radical right, specifically, and therefore more likely associated with the sort of politically controversial communications subject to hate speech suppression. For this, we employed Rydgren’s (2018, pp. 23–24) basic definition of the radical right which emphasises ethnonationalism anchored in narratives about the past, directed towards strengthening the nation, returning to traditional values, and establishing a localised, organic, and ethnically homogenous polity. For clarification, ‘radical right’ is an ideological classifier or political preference for which we employ Rydgren’s definition. The alt-right is a contemporary, US-based white nationalist ideological movement characterised by radical right ideology. ‘Far right’ is a broader ideological classifier than ‘radical right,’ yet which overlaps with the latter to a great extent. ‘White supremacist’ is a particular ideological position which is generally a component part of these frameworks.

From this basis, we made a sub-selection of the nine most popular channels (based on the number of subscribers) from Data & Society’s report, all of which are in English. The 15 most popular video clips (in number of views) were selected from each channel. Except for the control, this selection excluded the rare material that had no political content nor any connections to radical right narratives whatsoever. The selection was made during spring and summer of 2019, apart from the control channel, where the final selection was made in June 2021.

### History Time Corpus

We selected a control channel, also in English, assumed to contain very little hate speech, namely ‘History Time’ ( Kelly, n.d.). This channel exhibits a certain thematic and conceptual overlap with the other material due to its focus on ethnicities in conflict and narratives about the past.

### Stormfront corpus

For contrast we included sentences from the English-speaking white supremacist forum *Stormfront* ( Stormfront, n.d.; dataset Garciá-Pablos & Perez, n.d.). *Stormfront* is a far-right discussion forum which can be assumed to contain a high degree of explicit hate speech ( Costello & Hawdon, 2019).

## Results

### Automatic classification

Table 4 presents descriptive statistics of the model’s predictions for the sentences across all three corpora. On average the model identifies over 50% more hate speech in the alt-right transcripts (.111) than in the history-channel (.069), and even more in the *Stormfront* material (.159). Though the difference is less pronounced across the quartiles, the level of identified hate speech in the alt-right corpus is consistently nested between the other two corpora.

**Table 4.** Descriptive statistics of predicted hate in synthetic sentences and synthetic paragraphs.

	N	*M*	*SD*	q1 ^a^	q2 ^a^	q3 ^a^	>.5 (%) ^b^
**Synthetic sentences**							
Alt-right	54,853	.111	.192	.008	.027	.109	7
History	47,357	.069	.140	.005	.015	.057	3
Stormfront	10,795	.159	.200	.022	.078	.211	8
Difference (%) ^c^	16	60	37	69	80	90	133
**Synthetic paragraphs**							
Alt-right	7,887	.463	.296	.195	.414	.738	43
History	6,812	.342	.262	.120	.268	.521	27
Difference (%) ^c^	16	35	13	62	54	42	59

^a^ The respective quartile of scores given by the model.

^b^ Share of units that are predicted to be hate speech with a score >.5.

^c^ The relative change from ‘History’ to ‘Alt-right.’

The second part of Table 4 contains the descriptive statistics of the predicted hate on the paragraph level (described above) for the alt-right and history channel; the *Stormfront* data consists largely of short posts across different threads which prevents aggregation into paragraphs. With this approach the overall level of hate is pronounced; a much higher level of hate speech is identified across both corpora and the relative difference between the two is decreased. Still, the overall level of identified hate speech remains notably higher in the alt-right corpus ( *M* = .463, *SD* = .296; 43% were predicted to contain hate speech).

On average, the model noted 4,8 percentage points more hate speech in the *Stormfront* data than in the alt-right *YouTube* data (.159–.111); 4,2 percentage points more hate speech in the alt-right *YouTube* data than in the *History Time* data; and, finally, 9 percentage points more hate speech in the *Stormfront* data than in the *History Time* data. This is consistent with the expectation that the control, *History Time*, contains the least amount of hate speech, and that the *Stormfront* data contains the most amount of hate speech.

Since the *Stormfront* corpus was annotated for hate speech, we can confirm that our model missed 80% of those instances that were annotated for hate speech. At the same time, the model correctly identified 90% of the instances of non-hate speech.

### Qualitative close reading

We evaluated the outcome of the automated identification through a close reading approach. The material flagged as likely to be hate speech, as well as the low-probability material, were furthermore scrutinised in relation to our view on hate speech as well as the textual context.

A subsidiary category of proximate hate speech was used to identify videos, synthetic sentences, or paragraphs, which were not themselves possible to categorise as hate speech, but which nonetheless were clearly parts of broader acts of hate speech or narratives identifiable as such in connection to the discursive context. The synthetic sentences can for instance be considered proximate hate speech in relation to the *meso level* of paragraphs, and the paragraphs (or sentences) in relation to the *macro level* of the video clip as such (a distinction elaborated below).

An example of proximate hate speech defined in relation to overarching levels would be a statement about the lower IQ scores of sub-Saharan native tribes within the context of a video that, considered as a whole, purveys scientific racism. The statement as such, while precarious, could be neutral in another context, but is here auxiliary to a claim of racial inferiority on a higher level, and should therefore be categorised as hate speech.

It is not our intention to support any particular definition of hate speech, nor its implementation in a context of moderation or censorship, but rather to explore the various outcomes of such an implementation of a normative understanding of hate speech.

### Levels of hate speech

In our qualitative assessment, we examined three categories of material in detail. We ran synthetic sentences derived from the transcripts through the classifier, the outcome of which served to flag and identify synthetic paragraphs constituted by said sentences, as well as entire videos.

Three levels of contextual proximity were brought to bear both on the methodological framing of the material analysed, as well as in the qualitative analysis as such: sentences, paragraphs, and videos.

The sentences represent the *micro context,* and it is on this level that the classifier operates directly. As an example, in the micro context of a synthetic sentence certain concepts can amount to propositions expressing speech acts that match the definition of hate speech. The sentences can also express complex ideas that likewise match the definition, or be sufficiently close thereto, to be flagged as hate speech. The micro context in our analysis consists of the synthetic sentence and the immediate text fragments before and after the synthetic sentence.

The synthetic paragraphs on the level above function as the *meso context.* Here, sets of related statements are interpreted in a manner similar to how we approach the sentences.

Finally, the videos are the *macro context.* It should be noted that several additional levels above the videos implicitly will follow from this sort of classification, such as the channels themselves or the entire U.S. alt-right discourse of the late 2010s. In the qualitative evaluation, we did not consider these additional levels of a macro or meta context other than in the sense of identifying overarching well-known ideological anchor points such as the great replacement theory or scientific racism, when interpreting statements at the lower levels.

### Inspecting sentences

We analysed the top 1,000 and the bottom 10,000 synthetic sentences through close reading. This tenfold difference between the groups stems from the model predicting that hate speech sentences are greatly outnumbered and this group therefore needs to be scrutinised closer. Table 5 summarises the results.

**Table 5.** Results of a close reading on sentence and paragraph level.

Sample	Not hate speech		Hate speech					
			Total		Direct		Proximate	
**Sentence**								
Top 1000	728	(72.8)	272	(27.2)	177	(17.7)	95	(9.5)
Bottom 10,000	9950	(99.5)	50	(0.5)	13	(0.1)	37	(0.4)
**Paragraph**								
Top 124	88	(71)	36	(29)	17	(13.7)	19	(15.3)
Bottom 124	122	(98.4)	2	(1.6)	0	(0)	2	(1.6)

Note. Values in parenthesis indicate the percentage share of the subcategory out of the corresponding sample.

The outcomes here are strongly divergent. In the top category, we consider 27.2% of the sentences to be hate speech, either explicitly or indirectly. Of this set we considered only about a third as proximate hate speech through the qualitative analysis.

The bottom category is almost devoid of material categorised as hate speech. The result of the close reading is that only 13 sentences out of 10,000 carry that designation, and 50 (5%) in total are either explicitly or indirectly considered hate speech. Here, on the other hand, 37 (0.4%) of the designated set was considered proximate hate speech.

### Inspecting paragraphs

The evaluation of paragraphs also gave us a marked division between top and bottom ( Table 5, above). We scrutinised 124 paragraphs, each at the respective ends of the hate speech probability hierarchy. In the top, 17 were considered incorporating hate speech, or were as such designated hate speech. Nineteen paragraphs contained material proximate to hate speech or could as such be considered proximate hate speech, whereas the remaining 88 paragraphs were found to be neutral.

In contrast, the bottom set contained almost nothing that could be considered even proximate to hate speech. Two were placed in this category, while the remaining 122 paragraphs were designated neutral. In addition, the two paragraphs in the proximate category were not very strong examples of this intermediate category, with one (Supplementary Material, channel *7_RIT_66560*, line 70) containing a quite tacit allusion to an antisemitic sentiment, and the other (channel *6_BP_26276*, line 115) a veiled reference to race realism with regard to the video as broader context.

### Inspecting videos

The 14 videos whose transcripts contained the highest ratio of synthetic sentences likely to contain hate speech were qualitatively assessed in detail. Four of these were considered explicitly to incorporate hate speech. In these, hate speech was not connected to individual synthetic sentences, but to larger sections of information across the video. Four additional videos were categorised as considered proximate hate speech. By analysing content and presentation, we designated the remaining six videos as neutral. Nine of these have since been removed from the platform, after an update of YouTube’s guidelines in the Summer of 2019 ( YouTube, 2019; Hern, 2020).

The 14 videos whose transcripts contained the lowest ratio of synthetic sentences classified as probably containing hate speech were likewise qualitatively assessed in detail. Of these, none could be considered incorporating hate speech *per se.* Five could be classified as containing material proximate to hate speech or as such considered proximate hate speech in accordance with the above. The remaining nine were designated as neutral. Eight of these videos have since been removed.

## Discussion

A key implication of this study is the fact that hate speech narratives in our material seem to be tacitly constructed at more complex levels of discourse. Explicit acts of immediate hate speech are almost non-existent, something that partly is due to the nature of the material, which is intended to disseminate a point of view before a neutral or amicable audience. But the lack of explicit hate speech is likely also due to the creators’ awareness of moderation and suppression of hate speech.

Initial observations to this effect compelled us to approach the material through three levels of analysis, the micro, meso, and macro levels, which confirmed the tendency towards positions and narratives being indirectly structured at more complex levels of discourse. This is focused through the non-binary distinction between hate speech and proximate hate speech.

Similar studies generally use a binary approach (e.g., hate/non-hate) when annotating, which inevitably obscures a more nuanced characterization ( Vrysis et al., 2021) and entails certain issues of definition.

When we move from sentences towards paragraphs, we can see how the proximate hate speech category grows. Something designated as proximate hate speech essentially ‘points upwards’ towards more complex levels of discourse involving a broader sphere of meaning. Paragraphs with the highest number of proximate-flagged synthetic sentences are thus being indicated in this way, and the same holds for entire videos. The false positive rate (i.e., sentences, paragraphs, and videos flagged by the model as hate speech) is similar for sentences (72.8%) and paragraphs (70.1%), but lower for videos (42,8%; 6 out of 14).

The false negative rate does not reflect the same pattern. While false negatives fall when we move from sentences to paragraphs, of the set of videos with the lowest ratio of flagged synthetic sentences, all of 5 out of 14 (35.7%) were considered proximate hate speech. This is likely an artefact of the general character of the material as such which overwhelmingly tacitly affirms or reproduces narratives that can be categorised as hate speech.

This upwards indication also implies that any actual reception of hate speech narratives is mainly indirect. In other words, we are here dealing with something much more akin to Ellul’s notion of integration propaganda (or sociological propaganda) than agitprop ( Ellul, 1973, ch. 1.3, sect. ‘Propaganda of Agitation and Propaganda of Integration’). This would imply that our corpus exemplifies long-term consensus building approaches rather than explicit calls to action or concrete efforts of organisation.

### The top and bottom extremes

We illustrate the qualitative designation of proximate hate speech by giving two examples from the top and bottom categories of sentences, respectively, in relation to their associated meso and macro levels.

Synthetic *sentence* 301 in the bottom 5% category reads: ‘group’s average IQ and sub-Saharan Africans would also have some of the most conservative and racist societies based on their group’s’ (Supplementary material, tab ‘bottom 5%’). This sentence immediately signifies an attribution of a causal relationship between IQ scores and political tendencies. In the context of the radical right discourse on race, this sort of causal inference is common and immediately invokes the discursive attribution of essential racial characteristics we for instance find in the framework of scientific racism ( Farber, 2011, ch. 2). The sentence does not explicitly signify hate speech by any stretch of the imagination, but it immediately appeals to an essential association between race and intelligence and a factor of comparison between distinct racial groups.

When we look for more information on the meso level, i.e., the synthetic paragraph in which the sentence in question is embedded, we find an explicit juxtaposition between the IQ scores of Aborigines and sub-Saharan Africans and that of East Asians and Japanese, with an added emphasis on the association between a lack of understanding of ‘the intricacies of the world’ and the lower IQ scores. We also see more of the implied connection between IQ scores and political affiliation which evidently is a polemic against a posited correlation between racism and lower intelligence such as in this transcribed synthetic paragraph:

a person to not understand the intricacies of the world and this in turns makes him a racist well then there’s a lot of explaining to do that would then make Australian Aborigines on average the most politically conservative as well as racist leaning groups of people on earth according to the same survey and their groups average IQ and sub-Saharan Africans would also have some of the most conservative and racist societies based on their group’s average IQ so by nations East Asians and the Japanese in particular would be the most welcoming to foreigners as their median IQ places them as among the (Supplementary Material, tab ‘Paragraphs,’ line 2257).

In other words, in maintaining that Aborigines on average would be the most politically conservative as well as racist leaning group, the author is actually saying that Aborigines are the least intelligent people in the world, with the worst capacity to ‘understand the intricacies of the world.’ Thus, the synthetic sentence, at the micro level, is categorised as proximate hate speech since it immediately implies the propositional content at the meso level. This notwithstanding, neither the sentence nor the paragraph was flagged by the filter as likely to contain hate speech.

When we look at an example from the top-level sentences categorised as proximate hate speech, the situation is similar. Sentence 209 reads: ‘thousands of people from the Middle East pouring into Europe at least let people talk about their fears right he’ll talk.’ The immediate signification here has little to do with hate speech. It connects high levels of immigration to ‘fears,’ and vaguely implies that there are efforts to suppress discussion of these fears.

Looking to the meso level, i.e., the transcribed synthetic paragraphs, the latter point is emphasised, explicitly mentioning forceful discursive repression of such discussion, yet there is still nothing akin to hate speech here:

the problem is that I really am always suspicious when there are significant social problems that nobody can talk about when when facts become a problem to the discussion the discussion itself has turned cancerous quote unquote and speech yami no we look I mean if you want to [assuage] people’s fears about hundreds of thousands of people from the Middle East pouring into Europe at least let people talk about their fears right he’ll talk about the facts that give them concern but everybody gets screamed down and that is not a good sign here’s another example so forty years ago the Swedish (Supplementary Material, tab ‘Paragraph,’ line 376; *word within brackets added due to an error in the automatic transcription*).

However, on the macro level, i.e., in the context of the entire video, a fuller picture emerges. The ‘fears’ of the lower levels are here connected to claims of mass rapes specifically targeting European women, the intentional displacement of white populations through the mass immigration of high-fertility non-white population groups, all of which are also associated to issues of racial characteristics as well as Marxist and feminist collusions to ‘destroy the West.’

### Examples from the macro level

As additional comparison we give two examples from the macro level, one video each from the top and bottom categories. We begin with the video ‘Milo obliterates student’ from September 2016, randomised from our bottom 10% of videos, i.e., the 10% with the lowest number of sentences assessed as likely to be hate speech. Incidentally, the video has since been removed. The video presents a rather short monologue by Milo Yiannopoulos who laments what he perceives as a suppression of humour in the media. He connects this process to authoritarian policies and adds that he embraces politically incorrect humour since he simply finds such things as ‘AIDS,’ ‘Islam,’ and ‘trannies’ funny. The speaker is then interrupted by a person from the audience and responds with a few sentences which include the clause ‘fuck your feelings.’ We designated this video clip as not containing hate speech, either immediately or proximately, in relation to our definitions above. Criticising content moderation policies is obviously not hate speech, nor is incivility, and the admittedly demeaning approach to the groups in question does not fulfil the requisites of hate speech, notwithstanding any protected characteristics.

The video ‘What the Founders Really Thought about Race’ is randomised from the top 10% of videos. The video clip in question is from May 2017 and features a documentary-style presentation of the ‘actual’ views on race held by the U.S. founding fathers. This video is also removed. The presenter begins by disputing the idea that racial equality was not affirmed by the founding fathers and adds that black slaves were held by around 40% of the land-owning colonists and that a segregationist perspective was dominant. It is further argued that the founding fathers and influential early politicians, including Lincoln, desired to expel blacks from the United States, not least due to the widespread disgust over ‘miscegenation.’ The presentation sums up by arguing that different races build different types of societies and implies that the evident contemporary social ills can be connected to a divergence from these ostensibly traditional views of the founding fathers. During the close reading, we designated this video clip as containing explicit hate speech in accordance with the above definitions, in part due to the express usage of ‘miscegenation’ and the implicit racial supremacist message at the end.

### Comparative examples from top and bottom sentences

In the following, we give examples of sentences automatically classified as hate speech and non-hate speech. We compare twelve synthetic sentences from the top and bottom categories, respectively. Three from each category designated as hate speech or proximate hate speech (including immediate context fragments before and after), and three from each found to not contain hate speech. The selection is made in order of appearance in the set, i.e., the six sentences from the top category are the first hate speech and non-hate speech sentences encountered when the set is sorted by highest to lowest likelihood of containing hate speech. The opposite holds for the bottom category.

Hate Speech, Top 5% (Supplemental Material, tab ‘Sentence top 5%’)

1. for [the patriarchy] is what took us to space you just want build roads build roads it is what build the (line 2)
2. because Trump said pussy and they were fine with that grab [them] by the pussy but never mind about what rap (line 3)
3. conditions in black ghettos but what caused the ghettos here’s their answer white society is deeply implicated in the ghetto white (line 4)

Not Hate Speech, Top 5% (Supplemental Material, tab ‘Sentence top 5%’)

4. is a mosque don’t have any idea you want to guess an animal your basic bitch who is the vice president (line 8 (semi-duplicate on line 15))
5. ginger here come up here come up here come on stand here come on stand here anymore Ginger’s no wages limit (line 22)
6. 1978 a country that starts with a you utopia you went full retard man never go full retard what do you (line 35)

Hate Speech, Bottom 5% (Supplemental Material, tab ‘Sentence bottom 5%’)

7. there are deliberate policies to make us a minority anywhere we live policies intended to destroy us as a whole as (line 92)
8. can replace them (Jewish journalist) wrote America is tearing itself apart as an embittered quite conservative minority clings to power terrified at (line 95)
9. rights) activists who support everything that weakens the nation-state. This Western mindset and this activist network is perhaps best represented by [George Soros] (line 98)

Not Hate Speech, Bottom 5% (Supplemental Material, tab ‘Sentence bottom 5%’)

10. a trump presidency might signal a sea change with Brexit happening all the anti-globalist anti-globalization movements the sort of populist conservative (line 2)
11. conservatives going progressives and progressive go and conservative talk shows I think it’d be really interesting I was a bit annoyed (line 3)
12. Qatari government is so morally upright and ethical now you can bring on all sorts of conservative writers conservative people in (line 4)

Interesting to note here is that all the hate speech designated sentences or context fragments from the bottom set invoke the great replacement, with the latter two connecting with explicit antisemitism in their broader context. The great replacement hypothesis refers to the idea that native Western populations are being intentionally replaced by racial others, chiefly Muslims, a process often assumed to be the effort of a Jewish conspiracy ( Betz, 2018).

Whereas the great replacement hypothesis of the alt-right discourse is clearly framed as support for purported victims of an ongoing genocide and only designated as hate speech precisely in relation to its generally accepted and quite implicit connections to antisemitic organization, it is perhaps not surprising that allusions to replacement will generally not be flagged as forms of hate speech.

Sentence three invites a clear example of the contextual analysis behind the ‘proximate’ designation. This fragment connects to American Renaissance’s themes of white supremacy in its framework (the actual implication is that it’s absurd that ‘white society’ has anything to do with the emergence of ‘ghettos’ and that these are a fruit of inherent racial inferiority).

### Comparison between removed and unaffected video clips

Of the top-scoring videos, only two out of 14 (14.2%) were removed from YouTube after the material was collected. Both (100%) were designated as proximate hate speech. Of the remaining videos, four (33.3%) were designated as hate speech, two (16.6%) as proximately hate speech, and four (33.3%) as not containing hate speech.

Of the lowest-scoring videos, eight out of 14 (57.1%) were removed. Three of these (37.5%) were found to be proximate hate speech. Of the remaining six, only one (16.6%) was designated as proximate hate speech, and five (83.3%) was found not to contain hate speech.

By a quick assessment, if most of the videos removed after our data gathering were targeted due to perceived problematic content, it seems that our model is validated by the moderation policies of the platform. However, the significant gap between the number of videos removed in the respective categories is counter intuitive. Due to the small numbers involved, and the wide variety of potential factors behind channel or video removal, little can be concluded from this gap.

The number of views of the videos in question surprisingly stands in a clearly negative rather than positive relation to a video being removed. Almost all the removed videos in the top category had a significantly lower view count at the time of our data collection than the videos not removed, whereas one immediate assumption would be that higher view counts would increase negative attention and thus the likelihood of removal.

Thematically speaking there seems to be little difference between the two categories. Both the removed and remaining videos of the 10% top category engage with issues of mass immigration to Europe, feminism, gender issues, Islam, and race – all in approximately equal measure. Furthermore, several titles of the unaffected videos (‘The rape of Europe,’ ‘The Islamic state of Sweden’) are more obviously inflammatory than many of the removed clips (e.g., ‘Did Trump just save Western civilization?’ and ‘Response to *Contrapoints* on Degeneracy’).

## Conclusions

Our purposes with this paper were to 1) transparently account for the steps needed when utilizing automatic hate speech identification, including key challenges in the process, while in relation to alt-right hate speech specifically, and (2) to identify the challenges in identifying hate speech in popular alt-right YouTube videos.

Our study addresses the issue of the automatic identification of contentious speech acts with a particular focus on the high-context character of the meanings conveyed. In this approach, which combines a quantitative and a qualitative analysis, our study is novel since previous research has combined qualitative analyses with distant reading. The results obtained from this approach, not least the high false negative rates which became obvious through close reading, clearly indicate that effective and consistent identification of hate speech communication necessitates qualitative interventions by human reviewers to avoid arbitrary or misleading applications. Indeed, the comparatively low accuracy of the filters determined through our qualitative review of the automatic flagging implies possible limitations of studies which claim a consistently high accuracy of automatic identification, e.g., data chiefly characterised by explicit discourses, or the omission of high-context communications in favour of a binary reading based on keywords.

This commonplace binary approach tends to force the rationale for designating content as hate speech to be self-contained within the literal meaning conveyed. Otherwise, it will entail an arbitrary and misleading designation of high-context content as explicit hate speech in and of itself. Even studies which explicitly engage with this issue risk falling into this trap. Paasch-Colberg et al. (2021) make a point of going beyond the ‘hate/no-hate’ dichotomy and provide a useful analysis of various possible characteristics and rhetorical strategies of hate speech communications. Nevertheless, they employ a binary classification which requires the problematic signification to be self-contained within a narrowly delimited act of communication for the requisite of hate speech to be fulfilled.

A simple context-sensitive qualitative approach like ours can remedy this by bringing into focus the indirect character of many of these communications which also will tend to characterise a discursive landscape where moderation and censorship is intensifying. This, incidentally, also precludes much of the value of purely automatic approaches.

There are structural impediments which render effective unsupported automatic identification of high-context communications difficult in principle. Automatic identification or moderation cannot account for an evolving, complex context of often indirect signification, which is hardly feasible in practice even with much more advanced algorithmic systems.

In general, this study in detail exemplifies a process whereby automatic hate speech identification could be utilised effectively. We see that several methodological steps are needed to operate in concert for the outcome to be useful, with both highly technical quantitative processing and traditional qualitative analysis being vital to achieve meaningful results.

With particular regard to the alt-right YouTube material, the main challenge in terms of detection and precise identification of hate speech relates to the often tacit and indirect framing. Identification demands orientation in the broader discursive context and the adaptation towards indirect expressions renders moderation and suppression both ethically and legally precarious. In concert with this finding, we saw significantly more material being automatically classified as hate speech in the comparatively private and unmoderated far-right *Stormfront* forum in comparison with the YouTube material, yet here also, the false negative rate was high (see Stormfront, n.d.).

### Limitations and future directions

A challenge for the study was that the YouTube material consists of proficient speakers’ text where the avoidance of hate speech is a priority. In voiding suppression or banning from the medium, they use indirect and contextually dependent forms of hate speech, which is considerably more difficult to detect than the more overt forms of hate speech found on some discussion fora with less stringent rules of expression. Consequently, it is unsurprising that the accuracy of the model was not very high. At the same time, it is precisely these types of narratives which use concealment techniques, rhetoric, and indirect hate speech that we need to be able to better identify in contemporary online communication.

Natural Language Processing is a field that is developing quickly, and new methods and datasets continue to appear. In this study we have limited ourselves to a relatively small corpus and simple methods in part to demonstrate what can be done without a complicated or state of the art setup. In the future we hope to see more studies that combine the quantitative methodologies from Data Science with traditional hermeneutic analyses.

Finally, the relationship of our findings to Overton window issues is important. A comparison of our dated material (mainly 2015–2018) to both earlier and later sets, with an eye to the political background discourse, would provide data towards ascertaining the correlations between efforts toward discursive subtlety and both the creators’ assumptions of popular reception and the character of acceptable discourses, and the level and character of actual suppression.

## Data Availability

Top and bottom sentences and paragraphs with unique identifiers calculated from YouTube transcriptions. Supplemental data for this article can be requested from mailto:mika.hietanen@kom.lu.se.
