# Supplementary material for: Automatic Identification of Hate Speech – A Case-Study of alt-Right YouTube Videos
Source: F1000Res. 2024 Apr 23;13:328. [Version 1] doi: 10.12688/f1000research.147107.1 (PMC11316942; doi:10.12688/f1000research.147107.1)
Supplement: Peer review history

# Reviewer report: Reviewer response for version 1

**DOI:** 10.5256/f1000research.161256.r309593

**Published:** 2024-08-20

**Authors:** Surabhi Adhikari

**Affiliations:** 1 Columbia University, New York, USA

This article describes a study that aimed to automatically identify hate speech in YouTube videos associated with the alt-right movement. The researchers developed a machine learning model to classify text as hate speech or not, using a combination of three existing annotated corpora of tweets. They then applied this model to transcripts from selected YouTube channels, comparing results from alt-right channels to a control history channel and a white supremacist forum (Stormfront).

 The methodology involved several steps:

1. Gathering and combining annotated corpora
2. Text preprocessing
3. Training a Recurrent Neural Network (RNN) classifier
4. Applying the classifier to YouTube transcripts
5. Qualitative close reading of select parts

The researchers segmented the YouTube transcripts into synthetic sentences and paragraphs to apply the classifier. They found that the model identified more hate speech in the alt-right content compared to the control, but less than in the Stormfront forum. They also conducted a qualitative analysis to evaluate the model's performance and examine the context of identified hate speech.

 Study Design and Technical Soundness: The study design appears appropriate for an exploratory investigation into automatically identifying hate speech. The researchers acknowledge the experimental nature of their approach and its limitations. They use a combination of quantitative and qualitative methods, which strengthens the overall design.

 The technical aspects of the machine learning approach seem sound. The researchers experimented with multiple models before settling on an RNN, used proper train-test splits to prevent overfitting, and employed hyperparameter tuning. Their approach to segmenting the YouTube transcripts into synthetic sentences and paragraphs is creative and well-reasoned.

 The study design is appropriate and the work is technically sound. The researchers employed a well-structured approach combining machine learning techniques with qualitative analysis. Their use of multiple datasets, including a control group and a high-hate speech forum for comparison, strengthens the design. The technical aspects, including the development and application of the RNN classifier, are implemented with care and attention to best practices in machine learning.

 The paper provides sufficient details of methods and analysis to allow replication by others. The researchers offer clear explanations of their data sources, preprocessing steps, model training process, and application to YouTube transcripts. They also describe their approach to segmenting transcripts into synthetic sentences and paragraphs. While some additional details could enhance replicability, the core methodological components are well-documented.

 The statistical analysis and its interpretation are appropriate for this study. The researchers present relevant descriptive statistics of the model's predictions across different corpora. Their interpretation of these results is measured and takes into account the exploratory nature of the study. The analysis aligns well with the study's objectives and provides a solid foundation for understanding the performance of their hate speech detection model across different contexts.

Is the work clearly and accurately presented and does it cite the current literature?

Yes

If applicable, is the statistical analysis and its interpretation appropriate?

Yes

Are all the source data underlying the results available to ensure full reproducibility?

Yes

Is the study design appropriate and is the work technically sound?

Yes

Are the conclusions drawn adequately supported by the results?

Yes

Are sufficient details of methods and analysis provided to allow replication by others?

Yes

Reviewer Expertise:

Natural Language Processing

I confirm that I have read this submission and believe that I have an appropriate level of expertise to confirm that it is of an acceptable scientific standard.

# Reviewer report: Reviewer response for version 1

**DOI:** 10.5256/f1000research.161256.r298551

**Published:** 2024-08-12

**Authors:** Somaiyeh Dehghan

**Affiliations:** 1 Sabanci University,, Istanbul, Turkey

Summary: This paper addresses the critical task of automatically detecting hate speech, focusing on popular alt-right YouTube channels that utilize moderation-dodging techniques. The study is highly relevant given the increasing prevalence of hate speech online and the sophisticated methods used by certain groups to evade detection. The authors aim to contribute to the field by exploring binary classification (hate speech vs. non-hate speech) using a substantial dataset of 42,430 tweets derived from three publicly available, annotated hate speech corpora. The study employs both traditional and neural network-based classification methods, implemented through SciKitLearn and TensorFlow, respectively. The authors report their best results with an RNN (Recurrent Neural Network) classifier model, which is a notable finding given the complexity and context-dependency of hate speech.

 Relevance: The relevance of this study lies in its potential to improve the accuracy and robustness of hate speech detection systems, particularly in contexts where language and content are manipulated to avoid moderation. The work has significant implications for social media platforms, content moderators, and policymakers concerned with curbing the spread of hate speech while respecting free speech.

**Major Points:**

- **Lack of Clarity in Data Selection Criteria: **The authors mention that their training data was selected from three publicly available datasets but fail to provide any criteria or justification for this selection. It is crucial to understand why these particular datasets were chosen over others and how representative they are of the broader spectrum of hate speech. Without this information, the study's findings may lack generalizability.
- **Unclear Test Data Description: **The paper does not adequately describe the test data used to evaluate the models. A clear understanding of the test data is essential for assessing the validity and reliability of the results. The authors should specify whether the test data comes from the same datasets as the training data or if it was sourced independently. Additionally, details on how the test data was selected, including any preprocessing steps, should be provided.
- **Insufficient Explanation of Methods: **While the authors list the classification methods used (e.g., random forests, CNNs, RNNs), they do not provide sufficient details about the implementation of these methods. This is particularly important for the neural network models, where the architecture, hyperparameters, loss functions, and optimization strategies are critical to understanding the model's performance. Including this information would allow other researchers to replicate the study and assess its robustness.
- **Lack of Comparative Analysis: **The results of different classification methods (e.g., random forest, CNN, RNN) are not compared in detail. A comparative analysis is essential to determine which method performs best under specific conditions and why. This analysis could provide insights into the strengths and weaknesses of each approach and guide future research in this area.

**Minor Points:**

- **Moderation-Dodging Techniques: **The paper briefly mentions that the alt-right YouTube channels employ moderation-dodging techniques, but it does not explore this aspect in depth. A more detailed discussion on how these techniques impact the detection of hate speech could enhance the paper's contribution.
- **Presentation of Results: **The results section could benefit from a clearer presentation of the performance metrics for each model. Tables or graphs that compare the accuracy, precision, recall, and F1-scores of the different models would make the findings more accessible and easier to interpret.
- **Constructive Feedback: **The study addresses an important problem and makes a valuable contribution to the field. However, to enhance its impact, the authors should provide more detailed explanations of their data selection process, test data, and the specifics of their classification models. Additionally, a thorough comparative analysis of the results would strengthen the study's conclusions and offer more actionable insights for future research. By addressing these points, the paper could serve as a more comprehensive and reliable resource for those working on hate speech detection and related NLP tasks.

Is the work clearly and accurately presented and does it cite the current literature?

Yes

If applicable, is the statistical analysis and its interpretation appropriate?

Yes

Are all the source data underlying the results available to ensure full reproducibility?

Partly

Is the study design appropriate and is the work technically sound?

Yes

Are the conclusions drawn adequately supported by the results?

Yes

Are sufficient details of methods and analysis provided to allow replication by others?

Partly

Reviewer Expertise:

I am an NLP specialist currently working on a hate speech detection project. My research focuses on developing automated methods to identify and analyze online hate speech and target detection. My team and I have encountered similar issues and concerns to those addressed in this study. Our work aims to enhance the understanding and detection of hate speech to contribute to a safer and more inclusive digital environment.

I confirm that I have read this submission and believe that I have an appropriate level of expertise to confirm that it is of an acceptable scientific standard, however I have significant reservations, as outlined above.

# Reviewer report: Reviewer response for version 1

**DOI:** 10.5256/f1000research.161256.r309594

**Published:** 2024-08-10

**Authors:** Tommi Buder-Gröndahl

**Affiliations:** 1 University of Helsinki, Helsinki, Finland

This paper analyses hate speech (HS) in alt-right Youtube videos, by combining automatic and manual methodologies. The authors train an RNN classifier on prior manually labeled HS data from tweets, apply it to video transcripts from Youtube (synthetically divided into pseudo-sentences and pseudo-paragraphs), and inspect the results both quantitatively and qualitatively. Their main take-home message is that alt-right videos utilize indirect means of expressing HS on a higher contextual level than sentences. This constitutes a serious challenge for automatic classification schemes that are built for finding explicit HS in narrow contexts.

 Two main concerns arise based on the study's methodology and quantitative results. First, the authors chose an RNN classifier, after experimenting with simple machine learning techniques (random forests, logistic regression, and naïve Bayes) and CNNs. However, for many years already, the Transformer architecture has been the leading deep learning architecture in NLP – specifically, large pre-trained Transformer-based large language models (LLMs) such as BERT or GPT. It is therefore unclear (1) why the authors did not experiment on Transformers, and (2) why transfer learning on an LLM was not considered. The RNN does not seem to represent the contemporary state-of-the-art in text classification.

 Second, the numerical results (Table 4) are somewhat hard to interpret: it is unclear what they actually tell us about HS on alt-right videos, Stormfront, or the control History channel. Since we lack an established ground-truth on which parts of the alt-right videos or the History channel are genuine HS, it is hard to say if the results tell us more about the frequency of HS or about deficiencies in the RNN classifier.

 I do not believe that either concern is ultimately detrimental to the paper, since its main value lies in the close qualitative reading of HS. That being said, there is a predictable response to the authors' conclusions about the limits of automatic HS detection: high-level contextual information might indeed be unavailable for simple machine learning systems or even RNNs trained from scratch; but pre-trained LLMs might access it significantly better. Even though I do not believe that this reply is sufficient to remove the authors' worries, it deserves more consideration in future work.

 In terms of strengths, the most notable contribution of the paper is the close reading of video transcript results. As the authors note, prior work on automatic HS detection has tended to limit qualitative methods to distant reading. While close reading cannot achieve the same level of coverage, it is indispensable for properly understanding HS. The division of the analysis into three levels (micro, macro, meso) is also an important addition, especially given the contrasting results between levels. The hypothesis that alt-right influencers lean less on explicit HS and more on indirect expressions is intuitively valid, but the further empirical support provided for it in the paper is clearly valuable.

Is the work clearly and accurately presented and does it cite the current literature?

Yes

If applicable, is the statistical analysis and its interpretation appropriate?

Partly

Are all the source data underlying the results available to ensure full reproducibility?

Yes

Is the study design appropriate and is the work technically sound?

Yes

Are the conclusions drawn adequately supported by the results?

Yes

Are sufficient details of methods and analysis provided to allow replication by others?

Partly

Reviewer Expertise:

My current research concerns the interpretation of LLMs (and, more generally, any language-processing cognitive systems) in terms of linguistic and semantic theory. I have also worked on HS detection as well as the application of NLP in security-sensitive settings more generally.

I confirm that I have read this submission and believe that I have an appropriate level of expertise to confirm that it is of an acceptable scientific standard.

# Reviewer report: Reviewer response for version 1

**DOI:** 10.5256/f1000research.161256.r294951

**Published:** 2024-07-09

**Authors:** Richard Sear

**Affiliations:** 1 The George Washington University,, Washington, DC, USA

**Summary**

 This study presents 1) an automated hate speech detection method and 2) challenges in identifying hate speech used in popular moderation-dodging alt-right YouTube channels. The methodology of developing an automated classifier builds on the well-established field and uses pre-existing annotated corpora of tweets as training/test data for a standard NLP classification task pipeline. The final model selected is high-performing and uses an RNN architecture.

 To study the YouTube transcripts, the authors construct synthetic sentences and paragraphs out of "atoms" (7-word chunks) using a sliding window technique. A length of 7 was decided based on manual experimentation and inspection. Sentence scores were then aggregated into video-level scores.

 YouTube audio transcripts are taken from the 15 most popular clips (by view count) from the nine most popular channels (by subscriber count) in a set of channels filtered from Data & Society's Alternative Influence 2018 report using various established hate definitions. As controls, text from a history YouTube channel and a white supremacist forum were also analyzed.

 During qualitative close reading, researchers identified "proximate hate speech" as well as direct hate speech. Researchers examined text at the sentence level, paragraph level, and full video level. The upshot is that this analysis found relatively little explicit hate speech (likely due to awareness of moderators) and increasing proportions of proximate hate speech with more complex analysis levels (sentence to paragraph to video). The authors conclude that it is important to go beyond a hate/no-hate dichotomy or classifying content for hate speech without its context. Future directions for this work could include improved datasets (i.e. more up to date) and more sophisticated models.

**Concerns**

 The authors identify three openly available datasets (HSOL, HatEval, OLID) they used. They then selected a subset of these, but identified the criteria used to do so, which allows me to recreate their training dataset. However, neither the Tensorflow code used to train the model nor the model itself are linked, with the only specification for the final model being that it used an "RNN architecture". This is too generic for me to be able to accurately replicate the authors' model but could easily be solved by publishing the training code/final hyperparameters.

 On a broader level, I was a little concerned to see Twitter data being used to train a classifier that was ultimately used for YouTube transcripts (a very different writing style), but the authors appear to be aware of this mismatch and make several notes about ways their methodology accounts for it. As the authors note in their "Future Work" section, new data regularly becomes available which could solve this issue.

**Strengths**

 The study is thorough and identifies many widespread issues with the current state of hate speech detection, as well as showcases useful insights that come from the authors' detailed analysis of the data. I particularly liked the incorporation of multiple levels of close reading to both validate the automated results and gain a human understanding of how real hate speech appears on YouTube. Though this level of detailed analysis is perhaps not feasible for the sheer volume of potentially hateful online content that exists today, it shows the valuable insights that, when teams are able to do so, come from applying multiple methods in concert. I also thought the identification of "proximate" hate speech was both spot-on and extremely important for future work. As the authors note, a simple binary classification of hate speech will often fall short and lose non-literal or contextual information that is essential to analyzing how hate speech spreads online.

 Additionally, authors were communicative and forthcoming when I requested access to their dataset on Zenodo.

Is the work clearly and accurately presented and does it cite the current literature?

Yes

If applicable, is the statistical analysis and its interpretation appropriate?

Yes

Are all the source data underlying the results available to ensure full reproducibility?

Yes

Is the study design appropriate and is the work technically sound?

Yes

Are the conclusions drawn adequately supported by the results?

Yes

Are sufficient details of methods and analysis provided to allow replication by others?

Partly

Reviewer Expertise:

My research focuses on online hate and extremist speech. I study automated detection methods using NLP as well as the mechanisms of how it spreads between online communities at scale. My team has run into some of the same issues and concerns the authors address in this study.

I confirm that I have read this submission and believe that I have an appropriate level of expertise to confirm that it is of an acceptable scientific standard.
